# Atrial Fibrillation in Hypertrophic Cardiomyopathy: Is the Extent of Septal Hypertrophy Important?

**DOI:** 10.1371/journal.pone.0156410

**Published:** 2016-06-03

**Authors:** Kyoung-min Park, Sung Il Im, Eun Kyoung Kim, Sang-chol Lee, Seung-jung Park, June Soo Kim, Young Keun On

**Affiliations:** 1 Division of Cardiology, Department of Medicine, Samsung Medical Center, Sungkyunkwan University School of Medicine, Seoul 135–710, Republic of Korea; 2 Division of Cardiology, Department of Internal Medicine, Kosin University Gospel Hospital, Kosin University College of Medicine, Busan 602–702, Republic of Korea; Tokai University, JAPAN

## Abstract

Hypertrophic cardiomyopathy (HCM) is a cardiac disease associated with a high incidence of atrial fibrillation (AF). Recent studies have suggested that interventricular septum thickness may influence the risk stratification of patients with AF. We evaluated the effects of septal hypertrophy on morbidity and mortality in patients with HCM. Patients were followed for a median of 6.1 years and were divided into two groups according to the extent of septal hypertrophy. A total of 1,360 HCM patients were enrolled: 482 (33%) apical or apicoseptal, 415 (28%) asymmetric septal, 388 (27%) basal septal, 38 (2.6%) concentric, and 37 (2.5%) diffuse and mixed type. Ninety-two all-cause deaths and 21 cardiac deaths occurred. The total event rates were significantly higher for patients with HCM with more extensive septal hypertrophy (group A) compared to those with HCM ± focal septal hypertrophy (group B), regardless of type (p<0.001). Arrhythmias occurred in 502 patients, with a significantly higher incidence in group A than in group B (p<0.001). Among patients with arrhythmias, the incidence of AF was significantly higher in group A than group B (p<0.001). In univariate Cox analysis, a greater extent of septal hypertrophy (p<0.001), E/E´ ratio (p = 0.011), and mitral regurgitation grade (p = 0.003) were significantly associated with developing AF. In multivariate Cox analyses, a greater extent of septal hypertrophy [odds ratio (OR) 5.44 (2.29–12.92), p<0.001] in patients with HCM was significantly associated with developing AF. In conclusion, a greater extent of septal hypertrophy is an independent predictor of progression to AF in patients with HCM.

## Introduction

There is a high incidence of various arrhythmias in patients with hypertrophic cardiomyopathy (HCM). Previous studies indicated that there is a 20% lifetime risk for the development of atrial fibrillation (AF) in patients with HCM, with a prevalence as high as 40% in those older than 70 years. [1–5] AF affects quality of life and increases morbidity and mortality. [1,6] In addition, AF increases the risk of heart failure (HF) and hospitalization. A recent HCM population study found AF to be a strong predictor of mortality even after adjusting for established risk factors. Previous studies have suggested that the magnitude of left ventricular (LV) hypertrophy and obstruction of the LV outflow tract (LVOT) are associated with an adverse prognosis and increased risk of AF, [7–9] while other studies found no association. [1,4] Some echocardiographic markers of left atrial (LA) dysfunction correlate with AF in HCM. Recently, Avegliano et al. reported that LV hypertrophy location and the presence of a dynamic obstruction can affect the degree of diastolic dysfunction. Impairment was greater in patients with obstructive asymmetric HCM and markedly less in patients with apical involvement. [10] In contrast, Kim et al. reported that the overall survival rate in apical HCM was similar to that of asymmetric HCM. [11] However, there have been no previous reports on the relationship between the extent of septal hypertrophy in patients with HCM and the occurrence of AF events. Therefore, in this analysis, we sought to better characterize the occurrence of AF according to the extent of the hypertrophied septum in a large, single-center, referral cohort with HCM.

## Materials and Methods

### Study population

We retrospectively analyzed data from 1,712 adult patients diagnosed with HCM who were evaluated at the Samsung Heart Vascular Stroke Institute (Seoul, Korea) between 1994 and 2010. Data on patient demographics, comorbidities, echocardiographic data, laboratory studies, exercise testing, and medication use were collected at the time of the initial visit. Additional clinical and transthoracic echocardiography (TTE) parameters were assessed by a detailed review. Finally, we enrolled 1,360 patients according to our inclusion and exclusion criteria.

**Fig 1:**
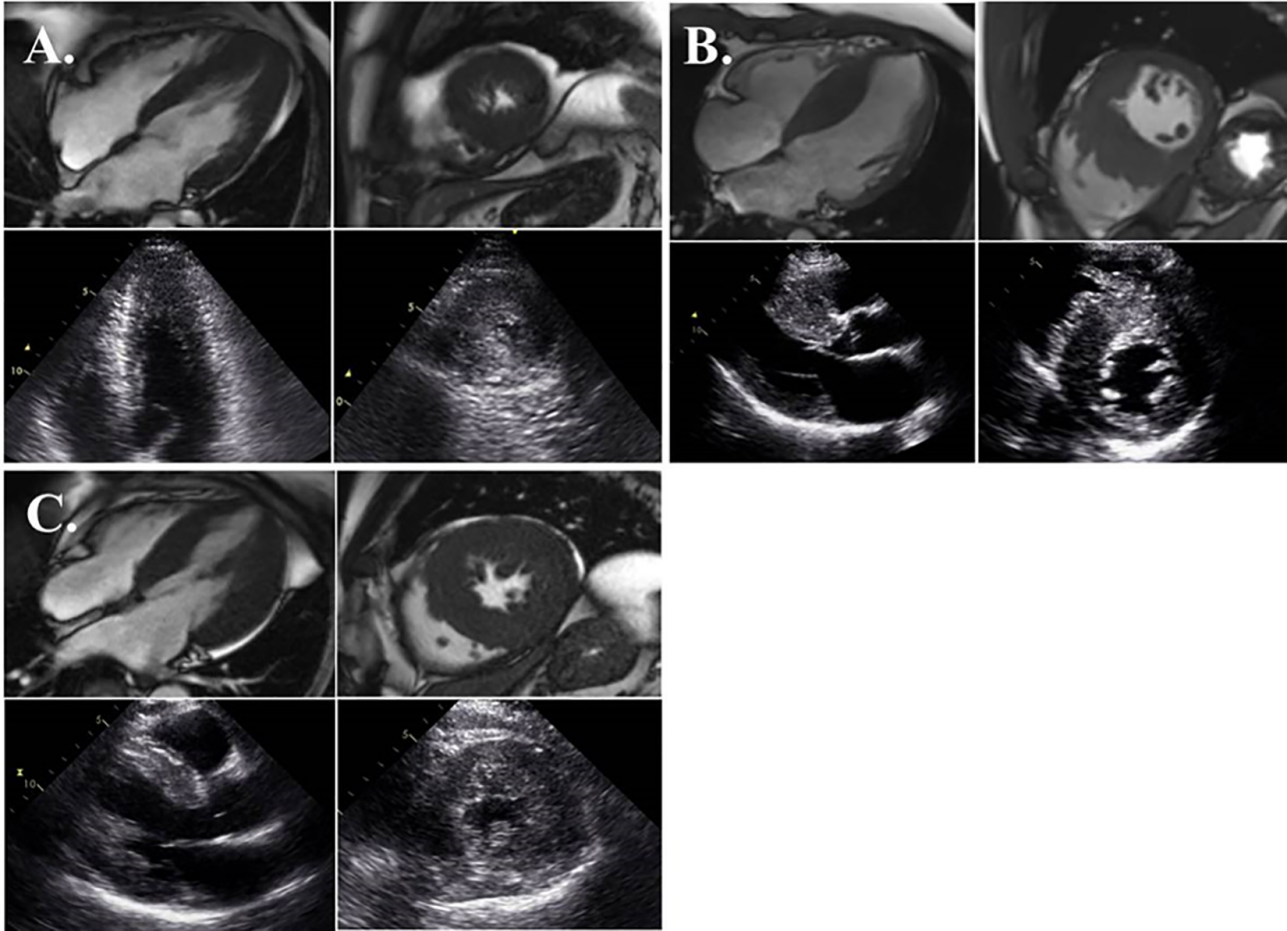
**Four- and two-chamber images (upper: MRI, lower: TTE) of each HCM type**: (A), apical; (B), septal; (C), diffuse.

**Fig 2:**
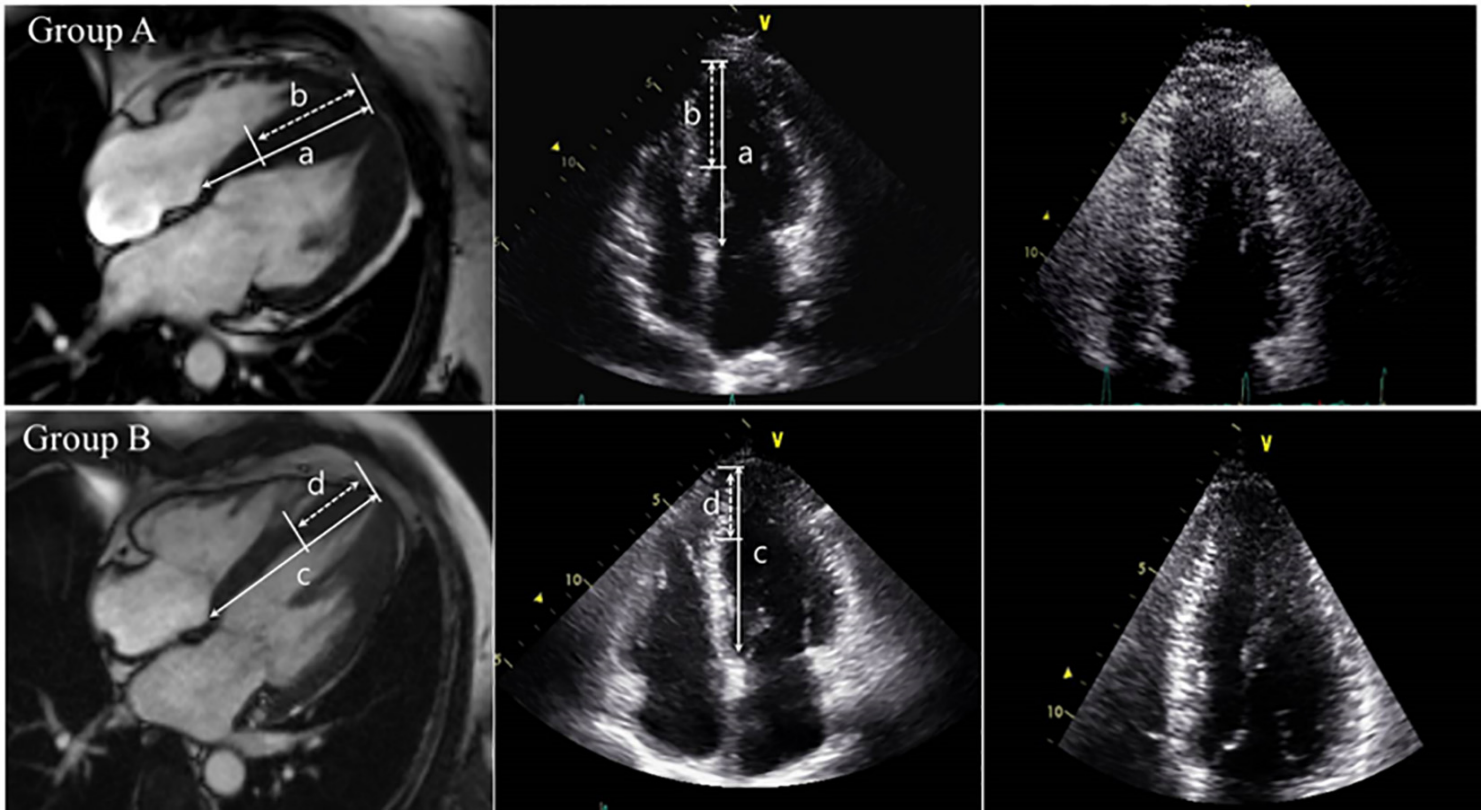
Reclassification of HCM involving the apex-septum according to our new imaging criteria. Group (A), HCM with more extensive septal hypertrophy (b/a >0.5); Group (B), HCM ± focal septal hypertrophy (d/c<0.5).

This study complies with the Declaration of Helsinki, and the research protocol was approved by the ethics committee of Samsung Medical Center. All patients provided written informed consent. The diagnosis of HCM was based on the echocardiographic criteria for HCM: 1) the absence of any underlying clinical condition that may lead to LV hypertrophy (i.e., long-standing systemic hypertension, aortic or subaortic stenosis, clinical evidence of metabolic storage disease or inflammatory disease); 2) end-diastolic LV wall thickness of 15 mm or more at any site or LV septal thickness with a posterior wall thickness of ≥1.3; 3) end-diastolic LV septal thickness with a posterior wall thickness of ≥1.5 (in patients with systemic hypertension); 4) LV hypertrophy confined predominantly to the LV apex (only the 4 apical segments and the apical cap according to the 17-segment guidelines of the American Society of Echocardiograhy) with a maximal apical wall thickness of ≥15 mm or a maximum apical-to-posterior wall thickness ratio of ≥1.3 during end-diastole regardless of the presence of systemic hypertension. [12,13] Each HCM was divided into one of three types based on two- or four-chamber TTE images and/or cardiac magnetic resonance imaging (MRI): A, apical (apical or apicoseptal); B, septal (asymmetrical septal or basal septal); or C, diffuse (concentric or mixed) type (Fig 1). Clinical characteristics and laboratory data for risk stratification of HCM were collected after diagnosis of HCM and were confirmed by echocardiography. Patients with a past history of atrial tachyarrhythmia (atrial fibrillation, atrial flutter, atrial tachycardia) or concomitant structural heart disease (e.g., myocardial infarction, severe valvular heart disease) were excluded. Of the patients with a past history of atrial tachycardia, 113 (59.8%) had septal-type HCM. We divided all patients into two groups based on morphology: HCM with more extensive septal hypertrophy (group A) and HCM ± focal septal hypertrophy (group B). HCM type was not considered in the group classification. If a patient had both a hypertrophied apex and a hypertrophied septum, they were regrouped according to the extent of disease in the septum. If septal hypertrophy extended more than half of the base-to-apex length of the septum (>0.5) on two- and four-chamber views, the case was placed in the group A. However, if septal hypertrophy was less than half of the base-to-apex length of the septum (<0.5) on two- and four-chamber views, the case was placed in the group B (Fig 2). In subgroup analysis, we divided all patients into two subgroups according to LVOT pressure gradient (≥30 mmHg or <30 mmHg) to compare differences in clinical outcomes. Baseline Holter monitoring was performed to detect arrhythmic events. Follow-up Holter monitoring was repeated at 6- and 12-month intervals or more frequently if palpitations were felt by the patient.

### Clinical outcomes

The primary outcome was AF events during the follow-up period. The secondary outcome was a composite of death from any cause, hospitalization for heart failure, cerebrovascular events (stroke, transient ischemic attack, cerebral hemorrhage, syncope), all arrhythmic events (AF, atrial flutter, ventricular tachycardia, ventricular fibrillation), and cardiac resynchronization therapy (CRT) or implantable cardiac defibrillator (ICD) implantation during the follow-up period. We conducted telephone interviews with the participants who could not attend regular follow-up. For any reported event, medical reports were retrieved and reviewed by physicians. We compared all clinical outcomes of HCM according to the type of HCM and LVOT pressure gradient.

### Echocardiography

TTE was performed using a commercially available system (Vivid 7, GE Medical Systems, Horten, Norway) with patients in the left lateral decubitus position. The LV ejection fraction was estimated using a modified Simpson’s method from the apical imaging planes. The left atrial volume was measured using the biplane modified Simpson’s method just before the mitral valve opening in the apical two- and four- chamber views, and the volume was adjusted to the body surface area (LAVI). The presence of an LVOT obstruction was evaluated via measurement of the LV pressure gradient from continuous-wave Doppler imaging. Dynamic LVOT obstruction was defined based on a LVOT pressure gradient (≥30 mmHg or <30 mmHg) on echocardiography with a Valsalva maneuver also noted. The early diastolic mitral annular velocity on the septal side (E´) and peak early diastolic trans-mitral flow velocity (E), E/E´, were calculated. All TTE measurements were reviewed by one of two authors (KMP or SII), who were blinded to patient outcomes. Assessment of inter-observer agreement was performed on a subset of 20 measurements. All TTE measurements were repeated five times. The mean of the five measurements was used for analysis to minimize the influence of measurement error.

### 24-hour Holter monitoring

Follow-up Holter monitoring was repeated at 6- and 12-month intervals and more frequently if palpitations recurred. All types of arrhythmias were detected on 24-hour Holter ECG monitoring and the date of documentation was recorded.

### Statistical methods

Pearson’s product moment correlation coefficient was calculated to quantify inter-rater variability in the measurement of both LV septal thickness and the distance of the hypertrophied septum. All continuous variables were expressed as either mean ± standard deviation or median (25th, 75th percentile range) depending on the distribution. For continuous data, statistical differences were evaluated using Student’s t-test or the Mann-Whitney U-test, depending on data distribution. To determine whether any of the variables were independently related to long-term clinical outcomes in patients with HCM according to HCM type, multivariate analysis of the variables with a p-value <0.05 in univariate analysis was performed using logistic regression analysis. The cumulative event proportions were calculated using Kaplan-Meier analyses. All correlations were calculated using Spearman’s rank correlation test. All statistical analyses were conducted using SPSS statistical software, version 19.0 (SPSS Inc., Chicago, IL, USA). The level of statistical significance was set at p<0.05 (two-sided).

## Results

### Baseline characteristics

In total, 1,712 patients were diagnosed with HCM during the study period. Based on TTE inclusion criteria, 82 patients were excluded. Thirty-eight patients had a history of myocardial infarction, and 12 had severe valvular heart disease. 112 were lost to follow-up. 108 patients were excluded due to a past history of atrial tachyarrhythmias and 65 (65/108, 59.8%) were septal-type HCM. Among these 108 atrial tachyarrhythmias, 78 patients were AF. Among these 78 patients, 61 patients met our TTE inclusion criteria and 42 patients (42/78, 56.4%) were classified in group A. Finally, we enrolled 1,360 patients diagnosed with HCM: 482 (33%) apical or apicoseptal, 415 (28%) asymmetric septal, 388 (27%) basal septal, 38 (2.6%) concentric, and 37 (2.5%) diffuse and mixed type (Fig 2). Of the 415 patients with asymmetrical septal HCM, 170 were reclassified as focal septal HCM according to our definition of ASH with more extensive septal hypertrophy. Among the 37 patients with mixed-type HCM, HCM was associated with the apex and free wall in 31, and with the apicoseptum and free wall in six patients. According to the extent of the hypertrophied septum, 558 patients were classified into group A and 802 patients into group B. The baseline demographic and echocardiographic characteristics of all patients are summarized in Tables 1 and 2. The mean age at the time that HCM was diagnosed, either at Samsung Medical Center or other institutes, was 54±17 years, and the majority of patients in this study were male (69%). The admission history due to congestive heart failure did not vary between the two groups (p = 0.559). In all, 2.6 percent of patients had a New York Heart Association (NYHA) Fc of III or IV. The distribution of NYHA Fc I-II was similar between patients in groups A and B. The number of NYHA Fc III-IV patients was significantly higher in group A than in group B (p<0.001). The mean N-terminal fragment of the prohormone B-type natriuretic peptide (NT-proBNP) level was 1933±6044 pg/mL. The mean LV septal thickness (mm) was 15.8±5.3, and the posterior wall thickness (mm) was 12.4±3.0. During the follow-up period (median 6.1 years, inter-quartile range 3.4 to 9.2 years) after diagnosis, 92 all-cause deaths and 21 cardiac deaths occurred. An ICD was implanted in 38 patients including 17 for secondary prevention and 21 for primary prevention. Cardiac magnetic resonance imaging (cMRI) was performed in 23% of all patients.

**Table 1:** Patient characteristics and medications according to HCM type.

Variables	Group A(n = 558)	Group B(n = 802)	p-value
Age (years)	61.3±16.8	63.0±17.4	0.072
Sex (male, %)	392 (70.3)	554 (69.1)	0.675
DM (%)	21 (13.9)	20 (11.4)	0.508
HTN (%)	66 (11.8)	75 (9.4)	0.911
CHF (%)	30 (5.4)	50 (6.2)	0.559
CAD (%)	35 (6.3)`	64 (8.0)	0.245
Syncope (%)	34 (6.1)	39 (4.9)	0.331
NYHA			
Class I	422 (75.6%)	639 (79.6%)	0.096
Class II	105 (18.8%)	159 (19.8%)	0.676
Class III-IV	31 (5.6%)	4 (0.6%)	<0.001
**Medications**			
Amiodarone	22 (4.2)	10 (1.4)	0.003
Beta-blocker	304 (59.4)	308 (45.4)	<0.001
Calcium channel blocker			
Verapamil	43 (8.4)	36 (5.3)	0.045
Diltiazem	67 (13.3)	76 (11.3)	0.322
ARB	106 (21.0)	172 (25.5)	0.083
ACEi	54 (10.7)	115 (16.9)	0.002
Furosemide	43 (8.5)	81 (12.0)	0.055
Spironolactone	25 (4.9)	43 (6.4)	0.315
Statins	49 (9.7)	109 (16.1)	0.001
Aspirin	140 (27.7)	230 (33.9)	0.023
Clopidogrel	19 (3.8)	48 (7.1)	0.015

Values are presented as mean±SD (range). HCM, hypertrophic cardiomyopathy; Group A, HCM with more extensive septal hypertrophy; Group B, HCM ± focal septal hypertrophy; DM, diabetes mellitus; HTN, hypertension; CHF, congestive heart failure; CAD, coronary artery disease; MI, myocardial infarction; TIA, transient ischemic attack; NYHA, New York Heart Association; ARB, angiotensin II receptor blocker; ACEi, angiotensin-converting enzyme inhibitor; MRI, magnetic resonance imaging.

**Table 2:** Echocardiographic findings according to HCM type at baseline.

Variables	Group A(n = 558)	Group B(n = 802)	p-value
**Echo parameters**			
LVEF (%)	62.8±8.6	65.2±12.0	0.010
LVIDs (mm)	27.2±5.2	30.4±8.8	< 0.001
LVIDd (mm)	45.7±6.2	49.8±8.2	< 0.001
IVSD (mm)	18.1±5.0	13.7±2.9	< 0.001
LVPWD (mm)	12.6±2.8	12.3±3.1	0.143
LAD (mm)	45.6±7.4	41.9±8.1	<0.001
LAVI (mL/m^2^)	57±15	44±17	<0.001
E velocity (cm/sec)	0.6±0.2	0.7±0.2	0.038
LV mass (g)	269.6±94.2	200±80.8	<0.001
LV mass index (g/m^2^)	149.6±38.7	117.4±41.6	<0.001
A velocity (cm/sec)	0.6±0.2	1.1±9.7	0.345
E/A	1.0±0.4	1.1±0.6	0.049
E/E’	13.9±6.7	11.6±4.2	0.034
MR grade	0.3±0.5	0.2±0.5	0.062

Values are presented as mean±SD (range). HCM, hypertrophic cardiomyopathy; Group A, HCM with more extensive septal hypertrophy; Group B, HCM ± focal septal hypertrophy; LVEF, left ventricular ejection fraction; LVIDs, left ventricular systolic diameter; LVIDd, left ventricular diastolic diameter; IVSD, interventricular septal thickness; LVPWD, left ventricular posterior wall thickness; LAD, left atrial diameter; LAVI, LA volume index; E, peak mitral flow velocity of the early rapid filling wave; A, peak velocity of the late filling wave due to atrial contraction; E’, early diastolic mitral annulus velocity; MR, mitral regurgitation; TR, tricuspid.

### Comparison between groups A and B

#### Patient characteristics

The baseline demographic and echocardiographic findings among 558 patients in group A and 802 patients in group B are summarized in Tables 1 and 2. The mean age and sex distribution were similar between the two groups. The past medical history of the patients did not differ significantly between the two groups. Beta-blockers and calcium channel blockers were used more often in group A than in group B. Angiotensin-converting enzyme inhibitors, statins, aspirin, and clopidogrel were prescribed significantly more in group B than in group A. Among laboratory findings, mean NT-proBNP level was significantly higher in group A (p = 0.010). Mean TSH level was not significantly different between the two groups (p = 0.727). Echocardiographic findings revealed that the LVEF was significantly lower for septal HCM (p = 0.010), and the LV dimensions were significantly greater for septal HCM (p<0.001). The LA dimension (mm) (p<0.001), LAVI (mL/m^2^) (p<0.001), LV mass (g) (p<0.001), and LV mass index (g/m^2^) (p<0.001) were significantly higher in group A than in group B. The E/A (p = 0.049) and E/E´ ratio (p<0.001) were significantly different between the two groups (Table 2). The degree of mitral regurgitation showed a trend toward being higher in group A (p = 0.062). cMRI was performed in only 23% (312) of all patients and significantly more frequently in group A than in group B patients (p<0.001).

#### Interobserver reliability of LV thickness measurements

The correlation coefficient for LV septal thickness was 0.861, and the distance of the hypertrophied septum was 0.887.

#### Clinical outcomes

**Fig 3:**
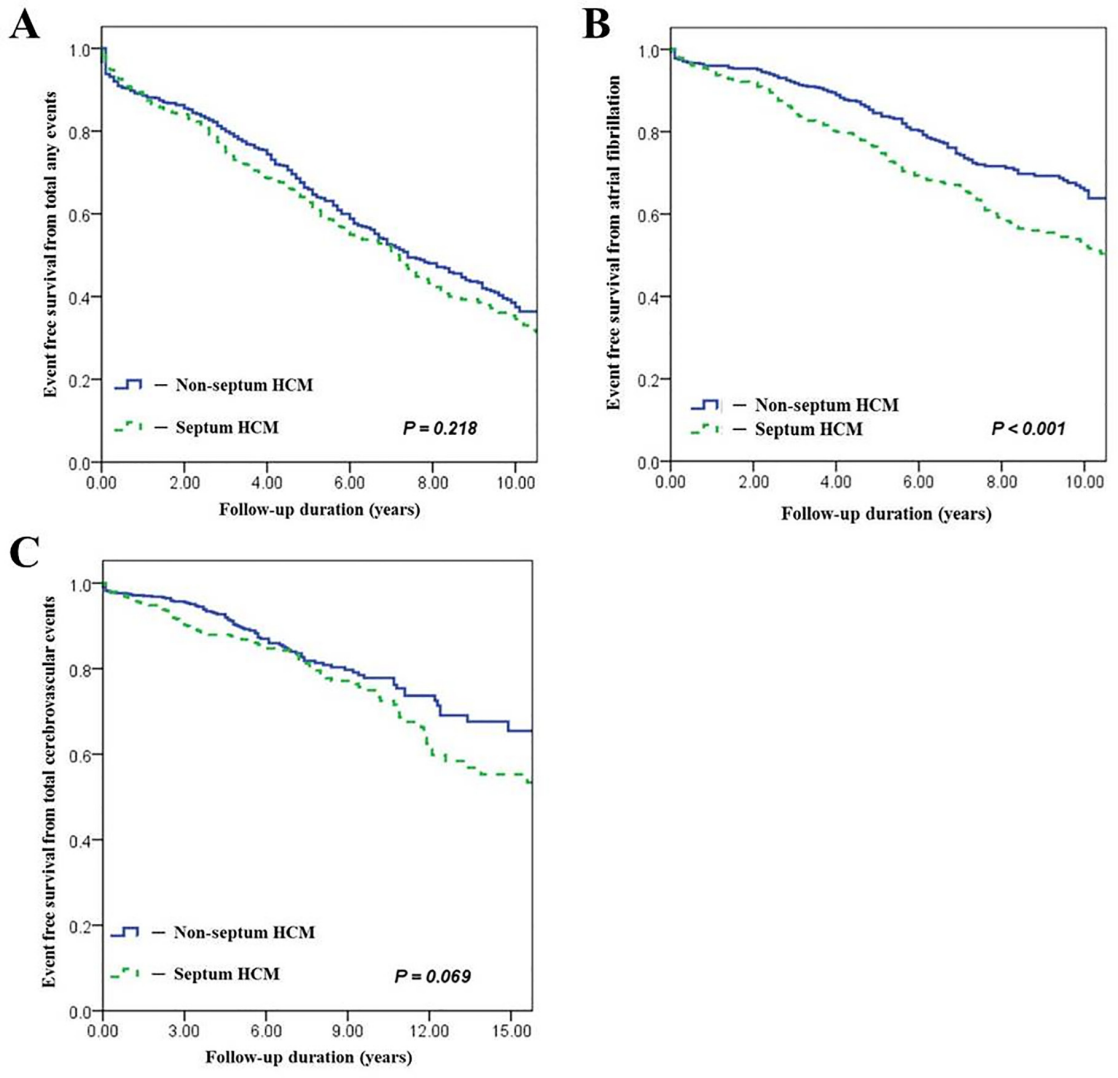
**Kaplan-Meier analysis of event-free survival in HCM patients according to the presence of predominant septal hypertrophy:** (A), all events; (B), atrial fibrillation; (C), all cerebrovascular events. HCM, hypertrophic cardiomyopathy.

There was no significant difference in the median follow-up period between patients in group A and group B after diagnosis (group A vs. group B; 6.3 years vs. 6.1 years, respectively; p = 0.235) in Table 3. The patients in group A experienced significantly higher total numbers of any events than those in group B (group A vs. group B; 58% vs. 44%, respectively; p<0.001). The cerebrovascular event rate (%) was significantly higher in group A (p = 0.004), and total arrhythmic events were also significantly more frequent in group A (p<0.001). Among clinical arrhythmias, the incidence of AF was significantly greater in group A than in group B (p<0.001). Among 281 AF patients, 50 (17.9%) patients had paroxysmal AF and 216 (76.8%) had persistent AF. The mean duration until first occurrence of AF after HCM diagnosis was 33.6±16.1 months. The incidence of persistent AF and permanent AF was significantly higher in group A (p<0.05). There were no differences in the mean duration until first occurrence of AF after HCM diagnosis in both groups (group A, 32.3±28.1 months vs. group B, 37.0±24.4 months, respectively; p = 0.702). The echocardiographic findings in AF according to HCM type at the 8-year follow-up indicated that the LV dimensions were significantly greater in septal HCM (p<0.001). LV mass (g) (p<0.001) and LVMI (g/m^2^) (p<0.001) were significantly higher in group A than in group B. Other parameters were not significantly different in both groups. All cause mortality rates were similar between the two groups (p = 0.380). The ICD implantation rate was significantly greater in group A than in group B (p<0.001). In univariate analysis, extensive septal hypertrophy (odds ratio (OR), 2.10; 95% CI, 1.65 to 2.68; p<0.001), E/E´ (OR, 1.049; 95% CI, 1.01 to 1.08; p = 0.011), and MR grade (OR, 1.55; 95% CI, 1.15 to 2.08; p = 0.003) were parameters significantly associated with AF (Table 4). In multivariate analyses, extensive septal hypertrophy was the only parameter that was significantly associated with AF (OR, 5.44; 95% CI, 2.29 to 12.92; p<0.001) (Table 4). In univariate analysis, a greater extent of septal hypertrophy was significantly associated with total event occurrence (OR, 1.58; 95% CI, 1.27 to 1.96; p<0.001) and cerebrovascular event occurrence (OR, 1.51; 95% CI, 1.11 to 2.04; p<0.008). Kaplan-Meier analyses indicated that only the AF-free survival rate was significantly lower in group A than in group B (Fig 3).

**Table 3:** Clinical outcomes according to HCM type.

Variables	Group A(n = 558)	Group B(n = 802)	p-value
Follow-up duration (months)	82.1±56.3	77.9±52.4	0.758
All events (%)	307 (55.0)	350 (43.6)	<0.001
Re-admission (%)	247 (44.3)	353 (44.0)	0.956
Cerebrovascular events (%)	97 (17.4)	98 (12.2)	0.009
Stroke (%)	39 (7.0)	42 (5.2)	0.200
TIA (%)	6 (1.1)	11 (1.4)	0.805
Syncope (%)	49 (8.8)	47 (5.9)	0.052
Cerebral hemorrhage (%)	47 (8.4)	52 (6.5)	0.203
Arrhythmic events (%)	280 (50.1)	222 (27.6)	<0.001
Atrial fibrillation (%)	147 (26.3)	134 (16.7)	<0.001
Paroxysmal	17 (11.5)	32 (23.8)	0.330
Persistent	120 (81.6)	98 (73.1)	<0.001
Permanent	10 (6.8)	4 (3.0)	0.027
Atrial flutter	27 (4.8)	28 (3.5)	0.263
Ventricular fibrillation (%)	14 (2.5)	10 (1.2)	0.095
Sustained VT (%)	25 (4.5)	16 (2.0)	0.010
Non-sustained VT (%)	67 (12.0)	34 (4.2)	<0.001
Death (%)	41 (7.3)	48 (6.0)	0.318
Cardiac death (%)	12 (2.1)	11 (1.3)	0.291
Non-cardiac death (%)	27 (4.8)	36 (4.4)	0.502
Unknown (%)	2 (0.3)	1 (0.1)	0.314
Device implantation (%)			
ICD (%)	31 (5.6)	7 (0.9)	<0.001
PPM (%)	5 (0.9)	13 (1.6)	0.336

Values are presented as mean±SD (range). HCM, hypertrophic cardiomyopathy; Group A, HCM with more extensive septal hypertrophy; Group B, HCM ± focal septal hypertrophy; TIA, transient ischemic attack; VT, ventricular tachycardia; ICD, implantable cardioverter defibrillator; PPM, permanent pacemaker.

**Table 4:** Univariate and multivariate Cox analysis of atrial fibrillation events.

	Univariate analysis		Multivariate analysis	
Variable, N (%)	OR (95% CI)	p-value	OR (95% CI)	p-value
Septum HCM	2.109 (1.655–2.687)	<0.001	5.447 (2.296–12.923)	<0.001
SEPT_THIC_ ≥30 mm	2.383 (0.912–6.224)	0.076	-	-
LVOT_PG_ ≥30 mmHg	0.724 (0.304–1.723)	0.465	-	-
Age, mean (years)	0.809 (0.655–1.687)	0.361	-	-
NYHA Class	1.002 (0.912–1.224)	0.486	-	-
LAD, mm	1.396 (0.604–1.923)	0.106	-	-
LAVI, mL/m^2^	1.461 (0.812–2.124)	0.088	-	-
E/A	0.918 (0.504–1.823)	0.315	-	-
E/E’	1.049 (1.011–1.089)	0.011		
MR grade	1.554 (1.159–2.083)	0.003		

OR, odds ratio; CI, confidence interval; HCM, hypertrophic cardiomyopathy; SEPT, septum; THIC, thickness; LVOT, left ventricular outflow tract; PG, pressure gradient; NYHA, New York Heart Association; LAD, left atrial dimension; LAVI, left atrial volume index; E, peak mitral flow velocity of the early rapid filling wave; A, peak velocity of the late filling wave due to atrial contraction; E’, early diastolic mitral annulus velocity; MR, mitral regurgitation.

## Discussion

The septum is a key component of ventricular interaction. There is a broad spectrum of experimental and clinical adverse effects in which septal dysfunction alters either left or right ventricular performance. Recently, Apostolakis et al. reported that interventricular septal thickness may influence the risk stratification of patients with AF. [9] The presence of AF is associated with more severe symptoms, worse exercise capacity, and a significantly higher risk of death from any cause, even after accounting for known risk factors of mortality in HCM and the use of antithrombotic, antiarrhythmic, and septal reduction therapies. [1,6] Several TTE parameters including higher LAVI, greater posterior wall thickness, higher E/E´ ratio, and shorter mitral deceleration time were found to be significantly correlated with the presence of AF.

In this retrospective analysis of HCM, approximately 20% of the study participants had a current or established history of AF at the time of index evaluation. Furthermore, HCM with more extensive septal hypertrophy was an independent predictor of arrhythmic events, especially AF. Moreover, the incidence of total cerebrovascular events was higher in patients with ASH with more extensive septal hypertrophy. There were some serologic and echocardiographic results that indirectly supported our data, such as NT-proBNP level, LA dimension/LAVI, and other TTE parameters, which reflected ventricular diastolic dysfunction.

We did not determine the precise point in time when patients developed AF (before or after diagnosis of HCM), nor the subsequent incidence of AF among patients in sinus rhythm during the index evaluation. However, the diagnosis of septal HCM has been shown to precede the development of AF in the majority of patients. [1] This finding strongly suggests that anatomical and physiological changes in the septum, including diastolic dysfunction, myocardial ischemia, and autonomic dysregulation, predispose patients to the development of AF. [14–16] LA enlargement is an established predictor of AF in HCM. [17,18] Consistently, a greater extent of septum involvement in patients with HCM is associated with a significantly larger LA dimension and higher LAVI compared to those with focal septal hypertrophy in our study. LA enlargement is a multifactorial process in HCM that depends on obstructive physiology, intrinsic myocardial stiffness, mitral regurgitation, and rhythm disturbances. [17] Previous studies in the general patient population clearly demonstrated an association between large LA and the risk of recurrent AF. [18,19] However, whether LA enlargement in HCM is a secondary phenomenon, a precipitator of AF, or a combination of these remains unclear. In this study, various echocardiographic parameters, such as the LA dimension/LAVI, E/E´ ratio, and NT-proBNP level, indicated that extensive septal hypertrophy might be related to poor clinical outcome, including total arrhythmic events and total cerebrovascular events. NT-proBNP is a functional cardiac marker produced primarily from cardiac ventricular myocytes in response to cardiac stress. Clinically, measurements of NT-proBNP are used in the diagnosis of LV systolic and diastolic dysfunction (cardiac stretch/stress) and as a prognostic marker for a variety of cardiac diseases. [20] In a previous study, Avegliano et al. revealed that the location of LV hypertrophy and the presence of a dynamic obstruction in HCM affect the degree of diastolic dysfunction; the same study also found that impairment is greater in patients with obstructive asymmetrical septal type disease, and markedly less in patients with apical involvement. [10] Lower E’ velocity, a significantly higher E/E’ ratio, and a clear increase in LA volume were also associated with a higher incidence of MR in this study. There were no significant differences in MR grade between groups. However, patients with a greater extent of septal hypertrophy had a larger LA size (LA diameter, LA volume index), higher LV mass index, and higher E/A, E/E’ ratio, which are representative of severe LV diastolic dysfunction. Consequently, we could infer that a greater extent of septal hypertrophy in patients with HCM can influence LA and LV diastolic function, which play important roles in the development of atrial arrhythmogenesis, However, further studies are required to demonstrate whether there are differences in the degree of fibrosis among the various morphological types and the relation to impairment in diastolic dysfunction.

Based on our results, we believe that the interventricular septum is an important structure associated with the development of various arrhythmias, especially AF. This result supports the importance of evaluating the extent of septal hypertrophy using TTE or cMRI after a diagnosis of ASH. If the extent is > 0.5, this may suggest to the clinician that close regular follow up is necessary for early AF detection and proper stroke prevention. To the best of our knowledge, this is the first study to divide the HCM patient population according to the extent of septal involvement of myocardial hypertrophy in order to compare various clinical outcomes. The median follow-up period was approximately six years. A large prospective study with a long-term follow-up is needed to confirm our results.

There are several limitations to our study. The reported associations should be interpreted with caution, as this was a retrospective approach using data obtained over a long period of time with an inherent risk of bias. We did not determine the precise point in time when patients developed AF (before or after diagnosis of HCM), nor the subsequent incidence of AF among patients in sinus rhythm during the index evaluation. Further prospective studies should be considered to validate our findings. This study has a selected population, and AF also presents in older age as demonstrated by the lack of young patients. Consequently, those who developed arrhythmias earlier in life could have been excluded. All Holter monitoring data were reviewed to obtain evidence of arrhythmic events, however it is possible that some arrhythmic events may have been missed. The analyzed population was derived from a single referral center and may represent a skewed HCM population with regard to disease severity and comorbidities. Because this was a referral cohort, many patients did not receive longitudinal care at our institution, which limited follow-up and data collection on the incidence of AF and cause of death. The cause-specific mortality analyses may therefore be underpowered to detect any associations. Data on stroke-related death were not available. The clinical outcomes reported in this study may also not be representative of the general population. cMRI is required to define the border of the hypertrophied septum, however in this retrospective study, cMRI was performed in only 23% of patients, 41% of whom were patients with apicoseptal HCM. Future studies should perform cMRI in all HCM patients to determine the influence of the extent of septal hypertrophy on clinical outcomes. In this study, the comparison was not based on wall thickness alone, and there may be important differences between groups that were not measured, such as LV mass. Extensive septal hypertrophy was only associated with AF in our study, and we could not provide an explanation as to why age and LA diameter were not associated with AF in this population. Finally, the definition of predominant septal hypertrophy used in this paper is highly subjective; however, we suggest that the extent of septal hypertrophy could be a simple useful predictor of progression to AF in patients with HCM.

## Conclusions

In this study, AF was associated with a higher incidence of septal involvement in patients with HCM, regardless of LVOT obstruction. In those patients, several TTE markers, including LA dimension, LAVI, E/A, and E/E´ were associated with AF, which represents diastolic dysfunction. Additionally, the patients with a greater extent of septal hypertrophy in HCM were more likely to develop AF than those with focal septal hypertrophy.

## Supporting Information

S1 File Long-term clinical outcomes and echocardiographic findings of HCM patients with AF according to HCM type on 8-year follow-up. (XLSX)

S1 Table Echocardiographic findings of HCM patients with AF according to HCM type on 8-year follow-up. Values are presented as mean±SD (range). HCM, hypertrophic cardiomyopathy; Group A, HCM with more extensive septal hypertrophy; Group B, HCM ± focal septal hypertrophy; LVEF, left ventricular ejection fraction; LVIDs, left ventricular systolic diameter; LVIDd, left ventricular diastolic diameter; IVSD, interventricular septal thickness; LVPWD, left ventricular posterior wall thickness; LAD, left atrial diameter; LAVI, LA volume index; E, the peak mitral flow velocity of the early rapid filling wave; A, peak velocity of the late filling wave due to atrial contraction; E’, early diastolic mitral annulus velocity; MR, mitral regurgitation; TR, tricuspid.
(DOCX)
