# Supplementary material for: Atrial Fibrillation in Hypertrophic Cardiomyopathy: Is the Extent of Septal Hypertrophy Important?
Source: PLoS One. 2016 Jun 3;11(6):e0156410. doi: 10.1371/journal.pone.0156410 (PMC4892478; doi:10.1371/journal.pone.0156410)
Supplement: S1 Table — Values are presented as mean±SD (range). HCM, hypertrophic cardiomyopathy; Group A, HCM with more extensive septal hypertrophy; Group B, HCM ± focal septal hypertrophy; LVEF, left ventricular ejection fraction; LVIDs, left ventricular systolic diameter; LVIDd, left ventricular diastolic diameter; IVSD, interventricular septal thickness; LVPWD, left ventricular posterior wall thickness; LAD, left atrial diameter; LAVI, LA volume index; E, the peak mitral flow velocity of the early rapid filling wave; A, peak velocity of the late filling wave due to atrial contraction; E’, early diastolic mitral annulus velocity; MR, mitral regurgitation; TR, tricuspid. (DOCX) [file pone.0156410.s002.docx]

**Supplemental Table. Echocardiographic findings of HCM patients with AF according to HCM type on 8-year follow-up**

| **Variables** | **Group A**  **(n=147)** | **Group B**  **(n=134)** | **p-value** |
| --- | --- | --- | --- |
| **Echo parameters** |  |  |  |
| LVEF (%) | 62.8±8.3 | 61.4±12.1 | 0.284 |
| LVIDs (mm) | 28.1±5.1 | 32.0±9.2 | <0.001 |
| LVIDd (mm) | 46.2±6.2 | 51.3±8.4 | <0.001 |
| IVSD (mm) | 18.1±5.0 | 10.8±2.8 | <0.001 |
| LVPWD (mm) | 10.9±2.8 | 10.4±2.5 | 0.086 |
| LAD (mm) | 49.0±7.0 | 47.8±9.9 | 0.245 |
| LAVI (mL/m^2^) | 56.0±12.4 | 54.8±14.6 | 0.354 |
| E velocity (cm/sec) | 0.7±0.2 | 0.8±0.2 | 0.236 |
| LV mass (g) | 278.9±106.1 | 210.2±77.5 | <0.001 |
| LV mass index (g/m^2^) | 149.8±36.8 | 120.5±40.6 | <0.001 |
| E/A | 1.2±0.7 | 1.4±0.9 | 0.316 |
| E/E’ | 14.3±5.9 | 13.3±4.9 | 0.260 |
| MR grade | 0.4±0.6 | 0.4±0.7 | 0.624 |

Values are presented as mean±SD (range). HCM, hypertrophic cardiomyopathy; Group A, HCM with more extensive septal hypertrophy; Group B, HCM ± focal septal hypertrophy; LVEF, left ventricular ejection fraction; LVIDs, left ventricular systolic diameter; LVIDd, left ventricular diastolic diameter; IVSD, interventricular septal thickness; LVPWD, left ventricular posterior wall thickness; LAD, left atrial diameter; LAVI, LA volume index; E, the peak mitral flow velocity of the early rapid filling wave; A, peak velocity of the late filling wave due to atrial contraction; E’, early diastolic mitral annulus velocity; MR, mitral regurgitation; TR, tricuspid.
